#  Disrupted Central Inhibition after Transcranial Magnetic Stimulation of Motor Cortex in Schizophrenia with Long-Term Antipsychotic Treatment

**DOI:** 10.1155/2013/876171

**Published:** 2013-03-14

**Authors:** Aulikki Ahlgrén-Rimpiläinen, Hannu Lauerma, Seppo Kähkönen, Ilpo Rimpiläinen

**Affiliations:** ^1^National Institute for Health and Welfare, Forensic Psychiatry, P.O. Box 30, 00271 Helsinki, Finland; ^2^Psychiatric Hospital for Prisoners, P.O. Box 49, 20251 Turku, Finland; ^3^BioMag Laboratory, Helsinki University Hospital HUCH, P.O. Box 340, 00029 Helsinki, Finland; ^4^Department of Clinical Neurophysiology, Helsinki University Hospital, P.O. Box 1020, 10601 Ekenäs, Finland; ^5^Institute of Biomedical Engineering, Tampere University of Technology, P.O. Box 553, 33101 Tampere, Finland

## Abstract

*Aims*. Schizophrenia is a neuropsychiatric disorder associated with mental and motor disturbances. We aimed to investigate motor control, especially central silent period (CSP) in subjects with schizophrenia (*n* = 11) on long-term antipsychotic treatment compared to healthy controls (*n* = 9). *Methods*. Latency and duration of motor evoked potentials (MEPs) and CSPs were measured with the help of single pulse transcranial magnetic stimulation (TMS) and intramuscular electrodes. After stimulation of the dominant and nondominant motor cortex of abductor digiti minimi (ADM) and tibialis anterior (TA) muscle areas, respective responses were measured on the contralateral side. *Results*. MEPs did not differ significantly between the groups. Multiple CSPs were found predominantly in subjects with schizophrenia, which showed a higher number of CSPs in the dominant ADM and the longest summarized duration of CSPs in the nondominant ADM (*P* < 0.05) compared to controls. *Conclusions*. There were multiple CSPs predominantly in the upper extremities and in the dominant body side in subjects with schizophrenia. Behind multiple CSPs may lie an impaired regulation of excitatory or inhibitory neurotransmitter systems in central motor pathways. Further research is needed to clarify the role of the intramuscular recording methods and the effect of antipsychotics on the results.

## 1. Introduction

Transcranial magnetic stimulation (TMS) of the motor cortex is a useful non-invasive method that enables investigation of motor cortex excitability and central inhibitory mechanisms in the central nervous system. The commonly measured parameters of the motor responses include motor conduction time (MCT), transient suppression of ongoing motor activity (central silent period = CSP), and latency and duration of CSP and motor evoked potential (MEP) amplitude. The lowest stimulus intensity that induces an appropriate response in the target muscle is considered to be motor threshold of the motor cortex [1]. 

The induction mechanism of the central silent period (CSP) is not completely understood. It has been suggested to consist of an initial part of spinal origin and of a later part of cortical origin [2–4] or to be of cortical, supraspinal origin, being generated in the primary motor cortex [5]. The CSP is probably controlled by complex subcortical extrapyramidal systems with numerous interneuronal synapses associated with (GABAergic) inhibitory circuits [6]. 

There are several TMS studies dealing with motor control in schizophrenia [7, 8], which is considered to be a neuropsychiatric disorder associated with mental as well as neurological symptoms, like extrapyramidal signs and neurological soft signs [9]. One of the earliest TMS studies [10] found that the latency of compound motor evoked potentials was significantly reduced in subjects with schizophrenia. Later TMS studies have not found significant differences in the motor threshold, motor conduction, MEP size, or cortical facilitation in schizophrenia compared to healthy controls [11, 12]. There is one study that reported that CSPs were divided into an early part with a weak suppression of voluntary EMG and a later component with a stronger suppression in patients with schizophrenia [13]. There is growing evidence for reduced cortical inhibition in nonmedicated subjects with schizophrenia [14, 15], but earlier TMS studies have shown a lot of variation of the results, especially when dealing with persons with schizophrenia on antipsychotic medical treatment [16–19].

The nigrostriatal, mesolimbic, and mesocortical dopamine pathways are the most important dopamine pathways, that modulate symptoms of schizophrenia. Through these pathways it is possible to induce desired therapeutic effects by antipsychotic treatment, but undesirable sideeffects are also possible. Glutamate (gamma-aminobutyric acid, GABA) has an important role in exciting the mesocortical pathways or inhibiting interneurons in the mesolimbic pathways [20]. Dopamine is one of the most important transmitters that influence the activity level in different brain regions. A dysregulation of dopamine interactions is supposed to result in positive or negative symptoms of schizophrenia, but interactions between dopaminergic and GABAergic neurotransmitter systems may play an important role in the pathophysiology of schizophrenia [20, 21].

Antipsychotic medical treatment relieves mental symptoms of schizophrenia, but conventional antipsychotics, like haloperidol, often cause sideeffects such as restlessness (akathisia), stiffness of body (rigidity) or movement disturbances (akinesia, dyskinesias), mainly by blocking dopamine (D2) receptors. Atypical antipsychotics, like clozapine, risperidone, and zotepine blockade, combined serotonin and dopamine (5HTC2/D2) receptors and induce less extrapyramidal side effects [20]. Earlier TMS studies have demonstrated convincingly that clozapine treatment has a lengthening effect on CSP in schizophrenia [22], probably due to its ability to interfere with serotonin (5HTC2)- and D2 receptors and GABA interneurons [20]. Abnormalities in CSPs may be connected with positive or negative symptoms of schizophrenia [21–23], but further research is needed. Abnormalities in CSPs have been found in various progressive neurological disorders often associated with movement disturbances, for example, Alzheimer's disease, stroke, Parkinson's disease, Huntington's disease, epilepsy, and motor neuron diseases like ALS [24]. In some neuropsychiatric disorders like unipolar depression [25], attention-deficit/hyperactivity disorder [26], and obsessive-compulsive disorder [27] the cortical inhibition has been found reduced. 

Because of the methodological diversity and the variation in the results of earlier TMS studies dealing with motor control in schizophrenia, we were challenged to investigate the basic motor control elements in schizophrenia, but by applying a different combination technique that has not been used in the earlier studies: intramuscular needle target electrodes and a single pulse TMS. We were interested to see if the TMS responses in subjects with schizophrenia on long-term antipsychotic treatment differed from controls or demonstrated side-to-side differences. We also wanted to see if the TMS parameters correlated with clinical parameters, like severity of current mental symptoms, movement disturbances, and the daily dose of antipsychotics. We expected no special findings in the motor conduction or in the motor evoked potentials, but we expected that the schizophrenia and control groups might differ from each other in the results of central inhibition.

## 2. Material and Methods

Inclusion criteria for volunteers with schizophrenia were ICD-10 diagnosis of schizophrenia (chronic, over one year duration of illness) and long-term (over one year) use of antipsychotic medication. Exclusion criteria (schizophrenia and control groups) were any comorbid (ICD-10 diagnosis) neurological or mental disease, a documented history of substance abuse or any serious traumatic injury of the extremities or brain or functional or neuroanatomic cerebral abnormalities. During the recent hospitalization, the participants with schizophrenia had been examined with computerized tomogram of head (CT) and electroencephalography (EEG). 

A total number of 11 volunteers from a hospital population (5 females, 6 males, mean age 42,6 years, sd = 13,7) with an ICD-10 diagnosis of schizophrenia (3 paranoid and 8 undifferentiated subtypes) and nine healthy volunteers from hospital personnel (5 females, 4 males, mean age 36,1 years, sd = 8,43) participated this study. Clinical examinations of subjects with schizophrenia were performed with the help of the following international psychiatric and movement disorder rating scales (Table 1): PANSS (Positive and Negative Syndrome Scale [28]), AIMS (Abnormal Involuntary Movement Scale [29]), SAS (Simpson-Angus Scale of extrapyramidal symptoms, [30]), BAS (Barnes Akathisia Scale [31]), and Calgary depression scale (depression associated with schizophrenia [32]) by two of the authors. 

The mean age of the subjects, the duration of schizophrenia and the mean daily dose and type of the used antipsychotic medication, categorized as atypical or conventional (in 100 mg chlorpromazine equivalents), are presented in Table 1. Four participants with schizophrenia were on clozapine treatment and one participant was using zotepine, which is an atypical antipsychotic (AA) agent like clozapine [20]. Other six participants with schizophrenia used combinations of conventional antipsychotics (CA), like perphenazine, thioridazine, and zuclopenthixol. Two CA users had additionally risperidone (mean daily dose: 8 mg, range: 4–12 mg), but because they showed clinically significant extrapyramidal signs, they were assessed to belong to the group of CA users. The mean daily doses of antipsychotics were converted to dose equivalents to 100 mg chlorpromazine (CPZ) [33, 34]. One AA user had additionally 3 mg of lorazepam/day and one CA user had 2 mg and one AA user 5 mg of lorazepam/day. All participants were asked to avoid taking lorazepam at least 2 days and any other psychotropic agents during a week prior to the TMS investigation [35, 36]. 

With the help of the Edinburgh handedness inventory [37], the handedness of all the participants was clarified. Two CA users and two AA users were left handed. All controls were right anded. The footedness was also asked and because this correlated with handedness, the dominance of the brain part and the body-side was determined according to the obtained handedness.

It was voluntary to participate in this study. The participants were informed about the study and they were required to give their written informed consent prior to the study. The clinical investigations were performed at the Department of Clinical Neurophysiology Helsinki, University Hospital (Ekåsens Hospital), Finland. The study was approved by the local ethics committee.

TMS was performed with the help of a commercially available magnetic stimulator, Cadwell MES-10, supplied with a round coil that has an external diameter of 9 cm. A biphasic stimulation pulse with an intensity of 60 to 80% of the maximum capacity of the device was applied. The stimulation intensity constantly exceeded the motor threshold level. In each series of stimuli, altogether five consecutive stimuli were given with a time interval of 1 to 5 seconds. For the stimulation of muscles in the upper extremities, the center of the coil was placed at the midpoint between the upper tip of the earlobe and Cz of the 10-20 EEG system, corresponding to the temporal area, contralateral to the side of the recorded responses. The most optimal site for the coil to stimulate the muscles in the lower extremities was to have the center of it located in the central area close to Cz of the 10-20 EEG system. Adjustments of coil positions were made to achieve the most favorable site of stimuli of the respective muscles. The shaft of the coil was directed backwards.

The relaxation level in an inactive muscle and the inhibition of the muscle activity in the preactivated muscle were monitored. The responses were recorded with the Dantec Keypoint device by using a pair of monopolar needle electrodes that were inserted into the abductor digiti minimi (ADM) muscles in the upper extremities and tibialis anterior (TA) muscles in the lower extremities at a distance of 3 cm from each other. The cathode was positioned proximal to the anode. The intramuscular recording with needle electrodes enables the measurement of the high-frequency components of the muscular activity and a high number of single-motor units simultaneously and illustrates a sizeable portion of motor tract reaching to that definitive muscle. The intramuscular recording provides a possibility to measure several single-motor units simultaneously. This method gives a more precise picture of the suppression of the high-frequency components in the muscle compared to the surface electrode measurement that provides information from the compound motor activity of the muscle [38–40]. 

Recommendations for the optimal TMS technique, intensity, and muscle contraction were applied [3]. The optimal stimulus location was determined by mapping the primary motor area with the stimulating round coil until the best response according to the amplitude criteria was achieved in the target muscle by a constant stimulus intensity that was above the level of the motor threshold. TMS was performed on the motor cortex of the dominant and the nondominant hemispheric areas. Because there were left-handed (*n* = 4) and right-handed (*n* = 5) subjects in the schizophrenia group, the calculations of all the TMS responses were based on the hemispheric dominance on the subjects. 

To measure the motor distal latency (MDL) and the latency of *F*-responses (*F*), electrical stimuli with rectangular pulses having a duration of 0.2 ms and intensities of 10 to 50 mA were given at the ulnar and peroneal nerves at the wrist and at the fibular head on the lateral side of the knee, respectively (Table 3). To measure *F*-waves, 10–20 stimuli were applied with a time interval of 1-2 sec, and the *F*-waves were identified. *F* was calculated from the minimum latency of the responses.

For the analysis of the muscle activation, the following parameters were recorded and calculated: (1) motor conduction time from cortex to ADM (MCTa) and to TA (MCTt), (2) MDL = respective motor distal latency to the stimulation of ulnar and peroneal nerves, (3) latency of *F*-response to the ulnar nerve stimulation at the wrist (*F*_*u*_) and to the peroneal nerve stimulation at the fibular head (*F*_*p*_), MDL was excluded from the *F* latency, (4) central motor conduction time from the motor cortex to the neck: CMCT_*n*_ = MCTa − (*F*_*u*_/2 + MDL), (5) central motor conduction time from the motor cortex to the lumbar area: CMCT_*l*_ = MCTt − (*F*_*p*_/2 + MDL). Based on studies with TMS of motor cortex, the MEPs show considerable amplitude variation [41, 42]. Therefore we did not find it relevant to assess amplitude sizes in this study. 

For the analysis of the CSP in each voluntarily maximally preactivated muscle, the following parameters were recorded on the contralateral side to a series of five magnetic stimuli: the latency, the duration, and the total number of the silent periods of the activated muscle (ADM, TA). The maximum preactivation of the muscle was defined as a full interference pattern of the muscle activity in a time frame of 500 ms and a sensitivity of 1 mV/div. The presence of the CSP was defined as a simultaneous decrease of amplitude of muscular activity below 0.05 mV/div in five consecutive measurements. Stimulation intensity was the same as that used to elicit MEPs.

For statistical analyses, PASW for Windows 18 was used. The values of the samples were not normally distributed, tested with the help of the Shapiro-Wilk test. The groups (i.e., schizophrenia and controls) were compared using the Mann-Whitney test for independent samples. Side-to-side differences within the groups (i.e., schizophrenia and controls) were analysed with the help of the Wilcoxon signed rank test for related samples. The schizophrenia group was divided due to antipsychotic medical treatment to two subgroups (i.e., users of AA and users of CA), but because of the small sample sizes the statistical analysis was not performed for the schizophrenia subgroups. The Kruskal-Wallis test for independent samples was used to test the possible age difference between the study groups (i.e., controls, users of AA, and users of CA). Spearman's correlation test was used to analyse the correlations within subjects of schizophrenia between TMS measures and PANSS, AIMS, SAS, BAS, Calgary, duration of schizophrenia, age of the subjects, and daily dose of the antipsychotics. The level of significance was set at *P* < 0.05. Bonferroni correction was used to correct the test results for multiple comparisons.

## 3. Results and Discussion

### 3.1. Characteristics of the Findings

All subjects completed the study protocol. The subjects with schizophrenia and the controls did not differ significantly on age (*P* > 0.05). Table 1 shows sociodemographic and clinical parameters in study participants.  Table 2 summarizes the mean values and the standard deviations (sd) of the main TMS results in all the study participants. The obtained *F*-waves and the motor distal latencies reflected the normal function of the peripheral nervous system (Table 3) in all study participants. 

**Figure 1:**
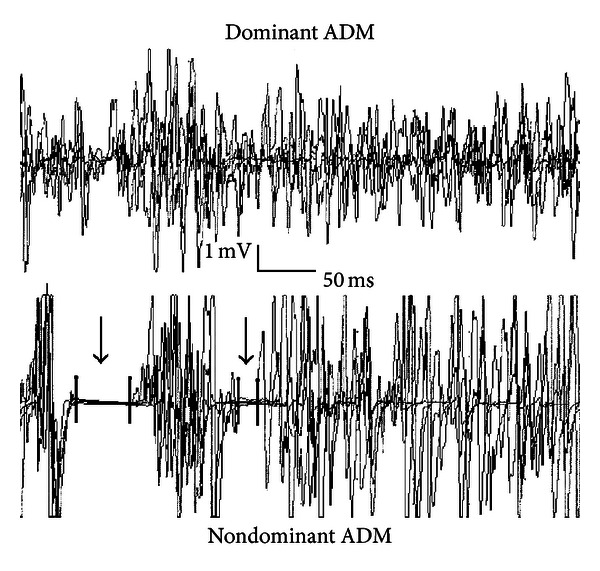
This figure demonstrates an example of an inhibitory respons in a subject with schizophrenia, who had clozapine medication. After TMS on the nondominant motor cortex of this subject, there could be observed 2 separate CSPs contralateral in the nondominant ADM (arrows pointing to the suppression of muscle activity). To compare, there are no inhibitory responses on the ipsilateral, dominant ADM.

Figure 1 presents a typical inhibitory response with multiple CSPs in the nondominant ADM of a subject with schizophrenia. The person was a clozapine user. Because it was observed that there are multiple CSPs predominantly in participants with schizophrenia and only in some controls, we decided to study the occurrence of multiple CSPs more closely and take it into account in the calculations. Eight out of 11 participants with schizophrenia had more than one CSP in the dominant and nondominant ADMs, whereas only two out of nine controls had more than one CSP in their ADMs. In the dominant ADM, the controls showed constantly only one compound of the CSP. In the nondominant TAs, four subjects with schizophrenia and three controls had more than one CSP, while in the dominant TA seven patients and only one control had more than one CSP. We measured the latency and duration of the first part of multiple CSPs (the first maximal suppression of the muscle activity). To calculate the total duration of the CSP, durations of the first and later occurring CSPs were added together in respective stimulation site.

### 3.2. Statistical Analysis

#### 3.2.1. Subjects with Schizophrenia versus Controls

*Dominant Site of Stimulation (Dominant Hemisphere/Dominant ADM and TA). *The schizophrenia group did not differ significantly from the controls in MCT, CMCT, or latency of CSP in ADM or in TA (*U* > 40.5, *P* > 0.05), but the schizophrenia group had a significantly higher number of CSPs in ADM (1.8 ± 0.6, *U* = 13.5, *P* = 0.004, significant even after Bonferroni correction, *P* = 0.024) and in TA (1.7 ± 0.6, *U* = 23.0, *P* = 0.046, but nonsignificant after Bonferroni correction). No significant differences were obtained in the first CSP duration or in the total duration of CSP in ADM or TA (*U* > 26.0, *P* > 0.05) between the groups (Table 2).

*Within Subjects with Schizophrenia*. The measured TMS parameters in ADM or TA (Table 2) did not correlate significantly with obtained records in PANSS, AIMS, SAS, BAS, Calgary, daily dose of antipsychotics, duration of illness or age of the subject (Table 1).

*Nondominant Site of Stimulation (Nondominant Hemisphere/Nondominant ADM and TA). *The schizophrenia group did not differ significantly from the controls in MCT, CMCT, or latency of CSP in ADM or in TA (*U* > 30.0, *P* > 0.05). The schizophrenia group had a higher number of CSPs in the nondominant ADM compared to controls (2.1 ± 0.9, *U* = 21.5, *P* = 0.03, but this was nonsignificant after Bonferroni correction, *P* > 0.05). Between the groups, no significant differences were obtained in the number of CSPs in TA or in the first CSP duration in ADM or TA (*U* > 25.0, *P* > 0.05). The total duration of CSP was significantly longer in ADM (166.9 ± 76.7, *U* = 15.0, *P* = 0.007, after Bonferroni correction even *P* = 0.049) in subjects with schizophrenia, but the groups did not differ from each other in the total duration of CSP in TA (*U* = 27.0, *P* > 0.05) (Table 2). 

In ADM, a positive correlation (rho = 0.791, *P* = 0.004, after Bonferroni correction even *P* = 0.04) were obtained between the number of CSPs (2.1 ± 0.9, Table 2) and PANSS (74,1 ± 15.1, Table 1).

*Within Subjects with Schizophrenia and Controls. *No significant side-to-side differences were observed within the study groups (i.e., schizophrenia and controls) in any of the measured TMS parameters.

*Within Subjects with Schizophrenia*. No significant correlations were obtained between the measured TMS parameters and the scores in PANSS, AIMS, SAS, BAS, and Calgary, daily dose of antipsychotic medication, duration of illness, or age of the subject.

*Between Users of Conventional Antipsychotics, Users of Atypical Antipsychotics, and Controls the following Tendencies were Observed. Users of CA* seemed to have the shortest mean values of the first CSP duration (ADM 72.0 ± 57.2/TA 48.3 ± 10.6) and total CSP duration (ADM 97.3 ± 81.6/TA 48.3 ± 10.6) in the nondominant extremities compared to controls and to the users of AA. 

*Users of AA* seemed to have the longest mean values of the first CSP duration (ADM 137.6. ± 85.6/TA 137.4 ± 75.7) and the longest total CSP duration (ADM 169.4 ± 102.0/TA 179.4 ± 88.6) in the nondominant extremities, but also in the dominant TA, where also the mean value of the number of CSPs was the highest (2.2 ± 0.5) of all (Table 2).

### 3.3. Discussion

The target of this study was to measure precise motor evoked potentials (MEP) and central silent periods (CSP) in the abductor digiti minimi (ADM) and tibialis anterior (TA) muscles in all four extremities of subjects with schizophrenia and compare the results to healthy controls. Single pulse transcranial magnetic stimulation (TMS) and intramuscular electrodes were applied. Clinical features of schizophrenia were taken into account when evaluating the results. 

The groups (schizophrenia and controls) did not differ from each other in respect of MEPs. Within the groups, there could not be demonstrated any significant side-to-side differences. These expected study results confirmed that the function of descending corticospinal motor pathways in schizophrenia was intact compared to healthy controls [11, 12].

However, our results indicate that central inhibition was disrupted into several separate compounds; in other words, recurrent CSPs could be observed mainly in subjects with schizophrenia. Contrary to the study by Davey et al. [13], the CSPs consisted of the first suppression of the EMG activity, followed by additional 1-2 weaker EMG suppressions that were observed mainly in the subjects with schizophrenia. After each TMS impulse, there could be up to one to three sets of inhibited muscular activity within 500 ms after the stimulation. 

We used an intramuscular pair of monopolar electrodes for recording, a technique that provides a possibility to record a high number of single-motor units simultaneously, thus, reflecting a comprehensive part of the function of the motor track to the target muscle. This is a more accurate method to define the behavior of muscular activity than the measurement of compound muscle activity with surface electrodes and enables the measurement of the high-frequency components of the muscular activity [38–40]. The changes in muscular electrical activity, including the firing frequency, can be evaluated, and the onset and end of the silent period can be easily and exactly determined. Thus, the use of the intramuscular electrodes might have helped us to detect all the compounds of CSPs, not only the first one, but also the later appearing, recurrent parts.

The statistically significant highest level of multiple CSPs occurred on the dominant upper extremities (ADM) in subjects with schizophrenia, but recurrent CSPs could be observed also in the nondominant ADM and dominant TA. Because high PANSS scores were linked to multiple CSPs (nondominant ADM), it is possible that there is a connection between regulation of central inhibition and mental symptoms of schizophrenia. The users of conventional antipsychotics seemed to show reduced central inhibition and more extrapyramidal signs like akathisia and abnormal involuntary movements; they were older, the duration of illness was longer, and their daily dose of antipsychotic medication was higher compared to the users of atypical antipsychotics (the differences were not statistically significant). The PANSS and SAS rates were about the same in both subgroups (Table 1). The users of atypical antipsychotics seemed to demonstrate increased and disrupted central inhibition, mainly in the nondominant body side and in the lower extremities. They also tended to have the longest CSP durations (Table 2), consistent with earlier studies, reporting that clozapine normalizes or even lengthens the reduced central inhibition in schizophrenia [22]. These findings and observations may reflect the severity of the current symptoms and the chronic course of illness of the study participants, especially on conventional antipsychotic treatment. A need for higher doses of conventional antipsychotics may in turn cause more neuroleptic-induced side effects. However, there are studies reporting modified CSPs in Parkinson's disease [43, 44] and decreased CSPs in restless legs syndrome (RLS) [45]. RLS and Parkinson's disease are supposed to have their pathophysiological origin in the disturbed basal ganglia that are responsible for the control mechanisms on the primary motor cortex [46]. Central dysregulation of dopamine transmission may not only promote psychiatric symptoms [47], but also play a role in causing motor changes. In our earlier studies we observed recurrent CSPs in nonmedicated subjects with chronic restless legs syndrome too [48]. Individuals with schizophrenia tend to exhibit a greater right-side than left-side parkinsonism caused by left-sided striatal hypodopaminergia unrelated to antipsychotic treatment [49] that may partly explain asymmetric findings in this study. We suggest that the physiological basics of multiple CSPs could be located in the extrapyramidal tracks and might reflect disturbances in their neurotransmitter systems or reflect interactions between GABA and dopamine regulation systems and that the findings in this study reflect not only changes in motor control in schizophrenia but also changes induced by antipsychotic medications [20]. 

Limitations of the study were the small study sample, a heterogeneity of the schizophrenia group, and left-handedness of some participants with schizophrenia. Because there were only three participants with paranoid schizophrenia, and because the two subtype groups (i.e., paranoid and undifferentiated schizophrenia) were mixed with users of atypical and conventional antipsychotics, we found it not possible to compare the results between the subgroups in a reliable way. Lorazepam and other additional psychotropic medications were set on a pause in good time before TMS investigations; thus, withdrawal symptoms were not probable.

## 4. Conclusions

The findings of our study can be summarized into three main aspects. (1) Descending corticospinal motor pathways and peripheral nerve systems were functioning correctly in schizophrenia. (2) Significant changes in the motor inhibitory system were observed in schizophrenia, seen as multiple central silent periods and their summarized lengthened outcome. (3) No significant side-to-side differences were observed within the subjects with schizophrenia or within the controls. The results may point to an impaired regulation of excitatory or/and inhibitory neurotransmitter systems in central motor pathways in schizophrenia. 

Further research is needed to clarify the role of the intramuscular recording methods and effects of antipsychotic medication on the TMS parameters. The same study procedure should be repeated in larger homogeneous schizophrenia populations with long-term antipsychotic treatment versus nontreated population.

## Figures and Tables

**Table 1:** Sociodemographic and clinical parameters of subjects with schizophrenia and controls.

Scales	PANSS	AIMS	BAS	SAS	AP (mg)	DUI	AGE	Subtype	Calgary
					(CPZ equiv.)	(years)	(years)	(*n*)	
Groups Control (*n* = 9)							36.1/		
*m*/sd							8.43		
Total (*n* = 11)	**74.09/ **	**7.36/ **	**1.18/ **	**1.27/ **	**638.84/ **	**17.27/ **	**42.09**	Paranoid (3)	**5.55** ± 2.8
*m*/sd	15.06	5.78	1.17	1.19	439.90	12.16	13.67	Undiffer. (8)	
CA (*n* = 6)	**73.83/ **	**10.67/ **	**1.83/ **	**1.33/ **	**700.00/ **	**22.50/ **	**49.50/**	Paranoid (1)	**4.83** ± 3.31
*m*/sd	17.53	5.96	1.17	1.51	496.5	12.63	12.45	Undiffer. (5)	
AA (*N* = 5)	**74.40/ **	**3.40/ **	**0.40/ **	**1.20/ **	**565.45/ **	**11.00/ **	**33.20/**	Paranoid (2)	**6.40** ± 2.07
*m*/sd	13.51	1.82	0.55	0.84	404.03	8.94	9.60	Undiffer. (3)	

Analysis between users of CA and AA (Mann-Whitney test)									

*U* test	14.5	4.5	4.5	13	11	5.5	4		8
*P* value	0.93	0.05	0.05	0.93	0.54	0.08	0.05		0.25

Analysis between controls, AA, and CA (Kruskal-Wallis test)									

Chi-square							5.94		
*P*-value							0.051		

Total: all subjects with schizophrenia; CA: subjects with schizophrenia, users of conventional neuroleptics; AA: subjects with schizophrenia, users of atypical neuroleptics; daily dose of antipsychotics (CPZ, mg in 100 mg chlorpromazine equivalents); DUI: duration of illness (in years) and age (in years) of the subjects with schizophrenia; *m*: mean value, sd: standard deviation; *n*: number of subjects; PANSS: Positive and Negative Syndrome Scale (total score); AIMS: Abnormal Involuntary Movement Scale; SAS: Simpson and Angus Scale for Extrapyramidal Signs; BAS: Barnes Akathisia Rating Scale (global clinical assessment of akathisia); undiffer./paranoid: undifferentiated/paranoid subtype of schizophrenia; Analysis: statistical analysis of the differences between the users of CA and AA (Mann-Whitney *U* test, *P*: probability, level of significance was set <0.05).

**Table 2:** The results of the TMS measurements and significant (*P*: probability) differences in the subjects with schizophrenia compared to controls.

Group	Controls	Total	CA	AA
Number	9	11	6	5
Site of stim.	ADM	ADM	ADM	ADM
	*m* (sd)	*m* (sd)	*m* (sd)	*m* (sd)
Nondominant body side				

MCT	21.5 (1.4)	21.2 (1.8)	20.7 (1.8)	21.8 (1.6)
CMCT	6.6 (1.2)	6.5 (1.5)	6.1 (1.3)	7.0 (1.6)
CSP				
First duration	104.2 (59.2)	101.8 (5.7)	72.0 (57.2)	137.60 (85.6)
Total duration	83.1 (57.8)	166.9 (76.7)	97.3 (81.6)	169.4 (102.0)
Total number	1.2 (0.4)	2.1 (0.9)	2.17 (1.17)	2.0 (0.7)
CSP latency	56.0 (7.6)	53.2 (6.2)	54.5 (6.6)	51.6 (6.1)
Site of stim.	**TA **	**TA**	**TA**	**TA**
MCT	28.9 (1.5)	29.9 (2.3)	29.0 (2.4)	31.1 (1.6)
CMCT	11.7 (1.5)	12.8 (1.6)	12.6 (1.2)	12.9 (1.5)
CSP				
First duration	60.3 (47.8)	88.8 (67.2)	48.3 (10.6)	137.4 (75.7)
Total duration	73.9 (58.0)	138.7 (101.8)	48.3 (10.6)	179.4 (88.6)
Total number	1.3 (0.5)	1.4 (0.5)	1.0 (0.0)	1.8 (0.5)
CSP latency	63.8 (10.8)	67.3 (7.3)	64.8 (8.8)	70.2 (4.1)

Dominant body side				

MCT	21.8 (1.6)	21.4 (1.7)	20.9 (1.9)	21.9 (1.4)
CMCT	6.9 (1.5)	6.5 (1.3)	6.0 (1.5)	7.0 (1.1)
CSP				
First duration	83.1 (57.8)	140.1 (79.7)	137.2 (91.6)	143.6 (73.3)
Total duration	106.0 (60.6)	130.1 (94.4)	166.8 (79.4)	167.0 (82.8)
Total number	1.0 (0.0)	1.8 (0.6)	2.0 (0.6)	1.6 (0.6)
CSP latency	56.6 (7.0)	54.9 (9.4)	56.2 (10.7)	53.4 (8.6)
Site of stim.	**TA**	**TA**	**TA**	**TA**
MCT	29.5 (2.1)	29.0 (4.2)	27.5 (5.0)	30.9 (2.1)
CMCT	12.2 (1.5)	12.0 (3.7)	11.0 (4.9)	13.2 (1.3)
CSP				
First duration	71.2 (51.7)	96.3 (59.0)	73.2 (37.5)	124.0 (72.1)
Total duration	67.1 (53.3)	107.9 (88.8)	87.8 (59.3)	199.8 (113.9)
Total number	1.1 (0.3)	1.7 (0.6)	1.3 (0.5)	2.2 (0.5)
CSP latency	68.0 (8.0)	69.1 (8.8)	67.0 (9.5)	71.6 (8.2)

Mean: *m*; sd: standard deviation; MCT: motor conduction time; CMCT: central motor conduction time; CSP: central silent period; ADM: abductor digiti minimi and TA: tibialis anterior muscles; total: all subjects with schizophrenia; CA: users of conventional antipsychotics; AA: users of atypical antipsychotics; time values are means in milliseconds, *n*: number of subjects.

**Table 3:** 

Parameter Body side	*F* (ms)	*F* (ms)	MDL (ms)	MDL (ms)	*n*	*n*	*n*STIM	*n*STIM
	ND	DO	ND	DO	ND	DO	ND	DO
CTRL/ADM								
Mean	24,7	24,2	2,5	2,7	11,9	10,7	17,2	16,4
Max	29	29,1	2,8	3,3	19	20	20	20
Min	21,4	18,1	2,2	2,3	5	5	11	8
SCH/ADM								
Mean	24,5	25,1	2,5	2,4	9,7	11,3	15,6	17,8
Max	26,7	28,7	2,9	2,8	17	18	20	20
Min	22,3	22	2	2	3	6	9	8

Parameter Body side	*F*	*F*	MDL	MDL	*n*	*n*	*n*STIM	*n*STIM
	ND	DO	ND	DO	ND	DO	ND	DO

CTRL/TA								
Mean	26,1	26,7	4,2	4,2	11,2	8,1	16,9	19,3
Max	29,7	27,8	5,2	4,8	19	10	20	20
Min	24	23,8	3,6	3,4	5	5	12	17
SCH/TA								
Mean	25,9	25,4	4,2	4,4	9,6	9,4	18	19,1
Max	32,5	30	5	5,2	19	15	20	20
Min	24,3	23,8	3,3	3,6	4	3	13	15

The mean, minimum, and maximum records for *F*-waves (=*F*, in milliseconds) obtained (number of *F*-waves: *n*) in ADM (A: abductor digiti minimi) and TA (T: tibialis anterior) muscles after 8–20 stimuli (number of stimuli: *n*STIM) given to each nerve site.

The minimum latency of each F-wave was measured. ND: nondominant hemispheric site, DO: dominant hemispheric site, CTRL: control, SCH: subject with schizophrenia, MDL: motor distal latency.

## References

[1] Cantello R, Gianelli M, Civardi C, Mutani R (1992). Magnetic brain stimulation: the silent period after the motor evoked potential. *Neurology*.

[2] Chen R, Lozano AM, Ashby P (1999). Mechanism of the silent period following transcranial magnetic stimulation. Evidence from epidural recordings. *Experimental Brain Research*.

[3] Säisänen L, Pirinen E, Teitti S (2008). Factors influencing cortical silent period: optimized stimulus location, intensity and muscle contraction. *Journal of Neuroscience Methods*.

[4] Roick H, von Giesen HJ, Benecke R (1993). On the origin of the postexcitatory inhibition seen after transcranial magnetic brain stimulation in awake human subjects. *Experimental Brain Research*.

[5] Schnitzler A, Benecke R (1994). The silent period after transcranial magnetic stimulation is of exclusive cortical origin: evidence from isolated cortical ischemic lesions in man. *Neuroscience Letters*.

[6] Trompetto C, Buccolieri A, Abbruzzese G (2001). Intracortical inhibitory circuits and sensory input: a study with transcranial magnetic stimulation in humans. *Neuroscience Letters*.

[7] Haraldsson HM, Ferrarelli F, Kalin NH, Tononi G (2004). Transcranial magnetic stimulation in the investigation and treatment of schizophrenia: a review. *Schizophrenia Research*.

[8] Soubasi E, Chroni E, Gourzis P, Zisis A, Beratis S, Papathanasopoulos P (2010). Cortical motor neurophysiology of patients with schizophrenia: a study using transcranial magnetic stimulation. *Psychiatry Research*.

[9] Dazzan P, Murray RM (2002). Neurological soft signs in first-episode psychosis: a systematic review. *British Journal of Psychiatry. Supplement*.

[10] Puri BK, Davey NJ, Ellaway PH, Lewis SW (1996). An investigation of motor function in schizophrenia using transcranial magnetic stimulation of the motor cortex. *British Journal of Psychiatry*.

[11] Fitzgerald PB, Brown TL, Daskalakis ZJ, Kulkarni J (2002). A transcranial magnetic stimulation study of inhibitory deficits in the motor cortex in patients with schizophrenia. *Psychiatry Research*.

[12] Boroojerdi B, Töpper R, Foltys H, Meincke U (1999). Transcallosal inhibition and motor conduction studies in patients with schizophrenia using transcranial magnetic stimulation. *British Journal of Psychiatry*.

[13] Davey NJ, Puri BK, Lewis HS, Lewis SW, Ellaway PH (1997). Effects of antipsychotic medication on electromyographic responses to transcranial magnetic stimulation of the motor cortex in schizophrenia. *Journal of Neurology Neurosurgery and Psychiatry*.

[14] Daskalakis ZJ, Christensen BK, Chen R, Fitzgerald PB, Zipursky RB, Kapur S (2002). Evidence for impaired cortical inhibition in schizophrenia using transcranial magnetic stimulation. *Archives of General Psychiatry*.

[15] Wobrock T, Schneider M, Kadovic D (2008). Reduced cortical inhibition in first-episode schizophrenia. *Schizophrenia Research*.

[16] Pascual-Leone A, Manoach DS, Birnbaum R, Goff DC (2002). Motor cortical excitability in schizophrenia. *Biological Psychiatry*.

[17] Fitzgerald PB, Brown TL, Daskalakis JZ, Kulkarni J (2002). A transcranial magnetic stimulation study of the effects of olanzapine and risperidone on motor cortical excitability in patients with schizophrenia. *Psychopharmacology*.

[18] Fitzgerald PB, Brown TL, Marston NAU (2004). Reduced plastic brain responses in schizophrenia: a transcranial magnetic stimulation study. *Schizophrenia Research*.

[19] Ziemann U, Tergau F, Bruns D, Baudewig J, Paulus W (1997). Changes in human motor cortex excitability induced by dopaminergic and anti-dopaminergic drugs. *Electroencephalography and Clinical Neurophysiology*.

[20] Stahl SM, Mignon L (2010). *Antipsychotics. Treating Psychosis, Mania and Depression*.

[21] Liu SK, Fitzgerald PB, Daigle M, Chen R, Daskalakis ZJ (2009). The relationship between cortical inhibition, antipsychotic treatment, and the symptoms of schizophrenia. *Biological Psychiatry*.

[22] Daskalakis ZJ, Christensen BK, Fitzgerald PB, Moller B, Fountain SI, Chen R (2008). Increased cortical inhibition in persons with schizophrenia treated with clozapine. *Journal of Psychopharmacology*.

[23] Wobrock T, Schneider-Axmann T, Retz W (2009). Motor circuit abnormalities in first-episode schizophrenia assessed with transcranial magnetic stimulation. *Pharmacopsychiatry*.

[24] Currà A, Modugno N, Inghilleri M, Manfredi M, Hallett M, Berardelli A (2002). Transcranial magnetic stimulation techniques in clinical investigation. *Neurology*.

[25] Bajbouj M, Lisanby SH, Lang UE, Danker-Hopfe H, Heuser I, Neu P (2006). Evidence for impaired cortical inhibition in patients with unipolar major depression. *Biological Psychiatry*.

[26] Richter MM, Ehlis AC, Jacob CP, Fallgatter AJ (2007). Cortical excitability in adult patients with attention-deficit/hyperactivity disorder (ADHD). *Neuroscience Letters*.

[27] Greenberg BD, Ziemann U, Corá-Locatelli G (2000). Altered cortical excitability in obsessive-compulsive disorder. *Neurology*.

[28] Kay SR, Fiszbein A, Opler LA (1987). The positive and negative syndrome scale (PANSS) for schizophrenia. *Schizophrenia Bulletin*.

[29] Munetz MR, Benjamin S (1988). How to examine patients using the abnormal involuntary movement scale. *Hospital and Community Psychiatry*.

[30] Simpson GM, Angus JW (1970). A rating scale for extrapyramidal side effects. *Acta Psychiatrica Scandinavica, Supplement*.

[31] Barnes TRE (1989). A rating scale for drug-induced akathisia. *British Journal of Psychiatry*.

[32] Addington J, Addington D (1999). Neurocognitive and social functioning in schizophrenia. *Schizophrenia Bulletin*.

[33] Lehman AF, Kreyenbuhl J, Buchanan RW (2004). The Schizophrenia Patient Outcomes Research Team (PORT): updated treatment recommendations 2003. *Schizophrenia Bulletin*.

[34] Sim K, Su HC, Fujii S (2009). High-dose antipsychotic use in schizophrenia: a comparison between the 2001 and 2004 Research on East Asia Psychotropic Prescription (REAP) studies. *British Journal of Clinical Pharmacology*.

[35] Ziemann U (2004). TMS and drugs. *Clinical Neurophysiology*.

[36] Di Lazzaro V, Pilato F, Dileone M, Tonali PA, Ziemann U (2005). Dissociated effects of diazepam and lorazepam on short-latency afferent inhibition. *Journal of Physiology*.

[37] Oldfield RC (1971). The assessment and analysis of handedness: the Edinburgh inventory. *Neuropsychologia*.

[38] Nandedkar SD, Sanders DB (1991). Recording characteristics of monopolar EMG electrodes. *Muscle and Nerve*.

[39] Barkhaus PE, Nandedkar SD (1994). Recording characteristics of the surface EMG electrodes. *Muscle and Nerve*.

[40] Barkhaus PE, Periquet MI, Nandedkar SD (2006). Influence of the surface EMG electrode on the compound muscle action potential. *Electromyography and Clinical Neurophysiology*.

[41] Hess CW, Mills KR, Murray NMF (1987). Responses in small hand muscles from magnetic stimulation of the human brain. *Journal of Physiology*.

[42] Rothwell JC, Thompson PD, Day BL (1987). Motor cortex stimulation in intact man. I. General characteristics of EMG responses in different muscles. *Brain*.

[43] Priori A, Berardelli A, Inghilleri M, Accornero N, Manfredi M (1994). Motor cortical inhibition and the dopaminergic system: pharmacological changes in the silent period after transcranial brain stimulation in normal subjects, patients with Parkinson’s disease and drug-induced parkinsonism. *Brain*.

[44] Ridding MC, Inzelberg R, Rothwell JC (1995). Changes in excitability of motor cortical circuitry patients with Parkinson’s disease. *Annals of Neurology*.

[45] Entezari-Taher M, Singleton JR, Jones CR, Meekins G, Petajan JH, Smith AG (1999). Changes in excitability of motor cortical circuitry in primary restless legs syndrome. *Neurology*.

[46] Ondo WG, Vuong KD, Jankovic J (2002). Exploring the relationship between Parkinson disease and restless legs syndrome. *Archives of Neurology*.

[47] Jarcho JM, Mayer EA, Jiang ZK, Feier NA, London ED (2012). Pain, affective symptoms, and cognitive deficits in patients with cerebral dopamine dysfunction. *Pain*.

[48] Ahlgrén-Rimpiläinen A, Lauerma H, Kähkönen S, Markkula J, Rimpiläinen I (2012). Recurrent csps after transcranial magnetic stimulation of motor cortex in restless legs syndrome. *Neurology Research International*.

[49] Caligiuri MP, Lohr JB, Jeste DV (1993). Parkinsonism in neuroleptic-naive schizophrenic patients. *American Journal of Psychiatry*.

